# Perspectives on program mis-implementation among U.S. local public health departments

**DOI:** 10.1186/s12913-020-05141-5

**Published:** 2020-03-30

**Authors:** Peg Allen, Rebekah R. Jacob, Renee G. Parks, Stephanie Mazzucca, Hengrui Hu, Mackenzie Robinson, Maureen Dobbins, Debra Dekker, Margaret Padek, Ross C. Brownson

**Affiliations:** 1 Prevention Research Center in St. Louis, Brown School, Washington University in St. Louis, One Brookings Drive, Campus Box 1196, St. Louis, MO 63130-4838 USA; 2 National Collaborating Centre for Methods and Tools, McMaster University, McMaster Innovation Park (MIP), 175 Longwood Road South, Suite 210a, Hamilton, Ontario L8P 0A1 Canada; 3 National Association of County and City Health Officials (NACCHO), 1201 Eye Street, NW, 4th Floor, Washington, DC 20005 USA; 4 Department of Surgery (Division of Public Health Sciences) and Alvin J. Siteman Cancer Center, Washington University School of Medicine; Washington University in St. Louis, 4921 Parkview Place, St. Louis, MO 63110 USA

**Keywords:** Implementation science, Mis-implementation, De-implementation, Evidence-based decision making, Evidence-based public health, Health departments

## Abstract

**Background:**

Public health resources are limited and best used for effective programs. This study explores associations of mis-implementation in public health (ending effective programs or continuing ineffective programs) with organizational supports for evidence-based decision making among U.S. local health departments.

**Methods:**

The national U.S. sample for this cross-sectional study was stratified by local health department jurisdiction population size. One person was invited from each randomly selected local health department: the leader in chronic disease, or the director. Of 600 selected, 579 had valid email addresses; 376 completed the survey (64.9% response). Survey items assessed frequency of and reasons for mis-implementation. Participants indicated agreement with statements on organizational supports for evidence-based decision making (7-point Likert).

**Results:**

Thirty percent (30.0%) reported programs often or always ended that should have continued (inappropriate termination); organizational supports for evidence-based decision making were not associated with the frequency of programs ending. The main reason given for inappropriate termination was grant funding ended (86.0%). Fewer (16.4%) reported programs often or always continued that should have ended (inappropriate continuation). Higher perceived organizational supports for evidence-based decision making were associated with less frequent inappropriate continuation (odds ratio = 0.86, 95% confidence interval 0.79, 0.94). All organizational support factors were negatively associated with inappropriate continuation. Top reasons were sustained funding (55.6%) and support from policymakers (34.0%).

**Conclusions:**

Organizational supports for evidence-based decision making may help local health departments avoid continuing programs that should end. Creative mechanisms of support are needed to avoid inappropriate termination. Understanding what influences mis-implementation can help identify supports for de-implementation of ineffective programs so resources can go towards evidence-based programs.

## Background

Mis-implementation of public health programs, policies, and services can occur in two ways: ending effective programs that should continue (inappropriate termination), or continuing ineffective programs that should end (inappropriate continuation) [1–3]. Here the term program refers to public health policies, environmental or system changes, educational and media activities, and services such as immunizations or screening for disease detection. De-implementation refers to ending ineffective or low-value programs, and is studied more often in medicine than in public health [1, 4–12]. The international Choosing Wisely initiative has recommended numerous medical procedures for de-implementation [13, 14]. McKay and colleagues (2018) recently outlined several public health and social service initiatives that have been discontinued or warrant de-implementation because they are harmful (prone infant sleeping position), ineffective (D.A.R.E. school-based drug prevention program), low value (routine HIV counseling with HIV testing), or the issue dissipated (Ebola) [15]. Evidence suggests these phenomena could have negative impacts on our public health systems [15].

In public health it is necessary to address both types of mis-implementation. Governmental public health departments in the US have experienced budget cuts in the past decade and high staff turnover [16, 17]. Finding ongoing funding is often challenging [15, 18–21]. For example, pressure to deliver programs within funders’ deadlines despite lack of funding for staff led to insufficient planning and incomplete statewide implementation of an evidence-based arthritis program [22]. External politics also influence funding and implementation decisions [21, 23]. Additional aspects influencing sustainment likely vary by program type and include implementation monitoring to improve program adaptation and delivery, partnerships, planning, and communications [18, 24–26]. Therefore, it is important to assess and address both types of mis-implementation in public health practice.

The impact of organizational supports for evidence-based decision making (EBDM) on mis-implementation of public health programs is not yet understood, though organizational structures and processes have been found to affect implementation of medical procedures and mental health services [7, 8, 20, 27, 28]. EBDM fosters implementation of effective programs and prevents mis-implementation through use of the best available surveillance data and intervention evidence in setting priorities and selecting programs, application of systematic prioritization and program planning methods, community engagement, and evaluation to inform adaptation and implementation [29–31]. Capacity building for EBDM includes training to increase skills of individual staff members and management practices to enhance organizational supports for EBDM.

A literature review identified five domains of organizational supports that are associated with agency performance: leadership, workforce development, organizational climate and culture, relationships and partnerships, and financial practices [32]. Leadership support for EBDM includes leadership skills, active modeling of EBDM processes, communication of expectations for use of EBDM processes, and participatory decision-making [32–34]. Workforce development includes in-service training and access to technical assistance. An organizational climate and culture supportive of EBDM has a free flow of information, values evidence and continued learning, and supports methods that may be new to the organization, such as specific prioritization or quality improvement processes [32, 33]. Relationships and partnerships with organizations from different sectors that align their missions and build EBDM capacity are essential, as no public health agency can accomplish the complex multi-level interventions in isolation [32]. Supportive financial practices are transparent and incorporate outcomes-based contracting and allocation of funds for quality improvement, EBDM, information access, and staff training; and diversify funding [32, 35, 36]. While these management practices support EBDM, little is known about direct relationships of these organizational supports with mis-implementation frequency.

To support use of EBDM and prevent mis-implementation, the Centers for Disease Control and Prevention (CDC) and other federal funders in the US increasingly require use of evidence-based interventions (EBIs) by grantees [37, 38]. In chronic disease prevention, CDC includes evidence-based practice requirements in funding state health departments that then pass on funding to local public health agencies. State health departments vary in the extent to which they in turn require local grantees to implement evidence-based strategies [33], even though they rely on local health departments (LHDs) to lead population health efforts to prevent chronic conditions [29]. Delivery of chronic disease prevention programs was not part of the historical regulatory responsibilities of LHDs and remains highly dependent on flow-through funds from federal and state agencies and private foundations. The Public Health Accreditation Board emphasizes workforce development for and documentation of evidence-based practice in its requirements for accreditation of state and local public health departments [39]. Public health workforce competencies include skills needed to plan and implement effective programs [40].

The purposes of the present study are to: 1) describe self-reported LHD frequency of and reasons for mis-implementation among a national sample of LHD chronic disease directors and 2) explore associations between perceived organizational supports for evidence-based processes and mis-implementation.

## Methods

### Study design

This national cross-sectional study was part of a larger study that used a stratified random sampling design to invite one individual from each included US LHD to complete an online survey [41]. Eligible LHDs were those that screened for diabetes or body mass index, or conducted or contracted for population-based nutrition and physical activity efforts, according to the 2016 National Association of County and City Health Officials (NACCHO) National Profile survey. Of the 1677 eligible LHDs, a total of 600 were randomly selected, 200 in each of three jurisdiction population size strata: small (< 50,000), medium (50,000-199,999), and large (≥ 200,000). The Washington University in St. Louis Institutional Review Board approved the study.

### Participants and data collection

The person responsible for making decisions about chronic disease prevention and control in each selected LHD was invited by email to complete the online Qualtrics survey. In some LHDs, this was the LHD director, while in other LHDs, this was a division director or program manager, according to lists provided by NACCHO. Invitees with invalid email addresses were deemed ineligible. Email invitations included materials for informed consent, as did the cover letter of the online survey. All participants gave online consent to the survey. Invitees could decline by email, phone, or online. Participants were offered a $20 Amazon.com gift card. To increase response rates up to three reminder emails were sent and two phone calls made to non-respondents. Data collection took place in August–September 2017.

### Survey development

As described in detail elsewhere, the study team drew from measures developed and tested in its previous national health department studies and other existing instruments identified by the study team [32, 41–43]. The survey included sections on mis-implementation (frequency and reasons), LHD and participant characteristics, skills for EBDM, and organizational supports. After three rounds of study team input and cognitive response testing with 10 chronic disease prevention practitioners, the survey demonstrated acceptable test-retest reliability [41].

### Measures

The four mis-implementation survey items included frequency and reasons for each type of mis-implementation. Frequency of mis-implementation included: “In your opinion, how often do programs end that should have continued (i.e., end without being warranted)”; and “In your opinion, how often do programs continue that should have ended (i.e., continue without being warranted). Response options were “never, rarely, sometimes, often, always, I do not know”. To understand reasons for mis-implementation, participants were asked, “when you think about public health programs that have ended when they should have continued, what are the most common reasons for programs ending”. And “when you think about public health programs that continued when they should have ended, what are the most common reasons for their continuation”. Response options included pre-listed reasons, “other,” and “I do not know.” The study team listed reasons found in the literature or commonly selected in pilot studies [2, 3]. In a pilot study with a purposive sample of chronic disease practitioners from LHDs, state health departments, and partnering agencies in multiple states, frequency of mis-implementation had 79% test-retest agreement for programs ending that should continue and 80% agreement for programs continuing that should have ended [2]. LHD characteristics included items from the 2016 NACCHO National Profile Survey and items within the current study’s survey. Participant demographic items were from the study team’s previous surveys. The supplemental file contains the complete survey instrument.

Survey items also assessed organizational supports for evidence-based decision making (EBDM) using a 7-point agreement Likert scale with 1 = strongly disagree and 7 = strongly agree. Confirmatory factor analyses in M-Plus supported the study’s conceptual framework of six organizational support factors [41, 44]. Factor scores for each participant were calculated in M-Plus. Table 1 lists the factors and items, with exact item wording.

**Table 1:** Evidence-based decision making (EBDM) support factors and items

Factor^a^	Item wording
Awareness of EBDM (3-items)	1. I am provided the time to identify evidence-based programs and practices.
	2. My direct supervisor recognizes the value of management practices that facilitate EBDM.
	3. My work group/division offers employees opportunities to attend EBDM trainings.
Capacity for EBDM (7-items)	1. I use EBDM in my work.
	2. My direct supervisor expects me to use EBDM.
	3. My performance is partially evaluated on how well I use EBDM in my work.
	4. My work group/division currently has the resources (e.g. staff, facilities, partners) to support application of EBDM.
	5. The staff in my work group/division has the necessary skills to carry out EBDM.
	6. The majority of my work group/division’s external partners support use of EBDM.
	7. Top leadership in my agency encourages use of EBDM.
Resource availability (3-items)	1. Informational resources (e.g. academic journals, guidelines, and toolkits) are available to my work group/division to promote the use of EBDM
	2. My work group/division engages a diverse external network of partners that share resources to facilitate EBDM.
	3. Stable funding is available for EBDM.
Evaluation capacity (3-items)	1. My work group/division plans for evaluation of interventions prior to implementation.
	2. My work group/division uses evaluation data to monitor and improve interventions.
	3. My work group/division distributes intervention evaluation findings to other organizations that can use our findings.
EBDM climate cultivation (3-items)	1. Information is widely shared in my work group/division so that everyone who makes decisions has access to all available knowledge.
	2. My agency is committed to hiring people with relevant training or experience in public health core disciplines (e.g. epidemiology, health education, environmental health).
	3. My agency has a culture that supports the processes necessary for EBDM.
Partnerships to support EBDM (3-items)	1. It is important to my agency to have partners who share resources (money, staff time, space, materials).
	2. It is important to my agency to have partners in healthcare to address population health issues.
	3. It is important to my agency to have partners in other sectors (outside of health) to address population health issues.

^a^Factors derived through confirmatory factor analyses by coauthor SM

### Statistical analysis

Data management, variable recoding, descriptive, and bivariate analyses were conducted in SPSS (IBM SPSS Statistics for Windows, Version 24.0) in 2018. Multivariate logistic regression modeling was conducted in SAS software (SAS Institute Inc., Version 9.4) in 2018. The two dependent variables, frequency of programs ending when they should have continued, and frequency of programs continuing when they should have ended, were each dichotomized into 1 = often or always and 0 = never, rarely, or sometimes, after excluding “I do not know” and blank responses. There were too few responses of “never” and “rarely” to analyze these responses as a separate categorical group. The n’s varied slightly because different numbers of participants answered don’t know. Separate modeling was conducted for each mis-implementation dependent variable and each organizational support for EBDM (the independent variable of interest in each model). All models were adjusted for LHD jurisdiction population size and state. Due to the study design with only one participant per LHD, and low intra-cluster correlation (ICC) statistics for the six EBDM support factors by state (which ranged from 0.005 to 0.012), mixed modeling was not conducted. Models were additionally adjusted for LHD or participant characteristics associated with both the mis-implementation frequency (dependent variable) and organizational support for EBDM (independent variable of interest).

## Results

### Participants

Of the 579 eligible LHD chronic disease leads with valid email addresses, 376 (64.9%) completed the online survey. Per the study design, there was only one participant from each LHD. Table 2 shows participant and LHD characteristics of the sample. Most participants were women (83.2%) and had worked in public health 10 or more years (71.4%). Participants had been in their positions an average of 6.5 ± standard deviation 6.5 years, with a median 4 years. The majority (58.2%) had a graduate degree; nearly a third (31.8%) had a graduate degree specifically in public health; and 29.1% had a nursing background. As designed, the stratified sample was split roughly in thirds by local health department jurisdiction population size. The sample included LHDs from 44 of 51 states (50 states and District of Columbia).

**Table 2:** Participant and local health department characteristics, by perceived mis-implementation, 2017 national survey

Characteristic	Overall *N* = 376^a^ %	Reported programs END that should have continued			Reported programs CONTINUE that should have ended		
		Often or Always (*n* = 106, 30.0%) %	Sometimes, Rarely, or Never (*n* = 247, 70.0%) %	Chi-square *P*-Value	Often or Always (*n* = 57, 16.4%) %	Sometimes, Rarely, or Never (*n* = 290, 83.6%) %	Chi-square *P*-Value
**Participants**							
Position				0.87			0.52
Agency leadership	46.4	46.2	48.6		43.9	49.0	
Program manager	45.6	47.2	44.1		45.6	44.5	
Technical or other	8.0	6.6	7.3		10.5	6.6	
Graduate degree in any field	58.2	51.9	62.0	0.08	68.4	57.9	0.14
Public health graduate degree	31.8	28.3	34.3	0.27	43.9	31.2	0.07
Nursing degree or license	29.1	34.9	26.4	0.11	21.1	29.5	0.20
Female	83.2	84.8	83.2	0.72	87.5	82.6	0.36
Age ≥ 50 years	43.7	41.5	45.1	0.53	31.6	45.3	0.06
Years worked in current position				0.76			0.94
< 5 years	54.0	50.9	52.8		54.4	52.9	
5–9 years	23.3	22.6	24.4		24.6	23.9	
≥ 10 years	22.7	26.4	22.8		21.1	23.2	
Years worked in public health				0.16			0.15
< 10 years	28.6	32.1	23.6		35.1	25.6	
10–19 years	31.6	33.0	32.1		35.1	31.5	
≥ 20 years	39.8	34.9	44.3		29.8	42.9	
**Local health departments**							
Jurisdiction population size				0.05			0.64
< 50,000	31.6	40.6	27.5		28.1	31.0	
50,000-199,000	34.3	30.2	36.8		40.4	33.8	
≥ 200,000	34.0	29.2	35.6		31.6	35.2	
Accredited^b^	28.0	23.6	30.0	0.22	29.8	29.0	0.90
Has a Local Board of Health	72.6	80.0	69.4	**0.04**^d^	73.7	72.2	0.82
Governance structure				0.09			0.26
Locally governed	76.3	76.2	76.1		75.4	76.1	
State governed	13.9	9.5	15.8		19.3	13.1	
Shared state/local governance	9.9	14.3	8.1		5.3	10.7	
Rural jurisdiction	45.6	51.4	42.5	0.12	40.4	44.6	0.55
Community Guide^c^ use to support decision-making in past year				0.73			0.87
Used consistently across all relevant program areas	6.0%	4.8%	6.5%		6.7%	6.4%	
Used in some program areas	63.5%	62.7%	65.1%		66.7%	63.0%	
Not used	30.5%	32.5%	28.5%		25.7%	30.6%	

^a^Mis-implementation n’s vary slightly because different numbers of survey participants answered “I do not know” or “not applicable”. *N* = 353 reported a frequency for programs end that should have continued. *N* = 347 reported a frequency for programs continue that should have ended

^b^Accredited by the Public Health Accreditation Board (PHAB), confirmed per PHAB list of accredited health departments

^c^Community Guide: Guide to Community Preventive Services, www.thecommunityguide.org/

^d^Boldface indicates statistical signifance (*p* < 0.05)

### Frequency of mis-implementation

Thirty percent (30.0%) of participants reported inappropriate termination, defined here as reporting programs often or always end that should have continued (Table 2). While frequency of inappropriate termination was not associated with any participant characteristics, those working in health departments with a jurisdiction population size < 50,000 were more likely to report inappropriate termination than participants in larger jurisdictions (*p* = .05). In addition, those in health departments governed by a local board of health were also more likely to report inappropriate termination compared to those with other forms of governance (*p* = .04). Only 16.4% reported inappropriate continuation, defined here as reporting programs often or always continue when they should have ended (Table 2). Frequency of inappropriate continuation did not differ by characteristics of participants or LHDs.

Table 3 shows adjusted odds ratios of reporting mis-implementation in separate models for each organizational support for EBDM. Organizational supports were not significantly associated with frequency of inappropriate termination in an unadjusted model and in a model adjusted for jurisdiction population size, state, having a local board of health, graduate degree in any field, nursing background, and years worked in public health. All six organizational support factors were negatively associated with inappropriate continuation after adjusting for LHD jurisdiction population size, state, public health graduate degree, and age group. That is, participants that reported higher presence of organizational supports for EBDM reported less frequent continuation of programs that should have ended.

**Table 3:** Adjusted odds ratios of reporting mis-implementation by organizational supports, in separate multivariate logistic regression models^a^

Perceived organization support factor	Reported programs often or always END that should have continued vs else (*N* = 353)^b^				Reported programs often or always CONTINUE that should have ended (*N* = 347)^c^			
	b (SE)	Wald	*P*-Value	Odds Ratio (95% CI)	b (SE)	Wald	*P*-Value	Odds Ratio (95% CI)
Awareness of EBDM	0.24	1.75	0.19	1.28 (0.89, 1.83)	−0.58	6.21	0.01	0.56 (0.36, 0.88)
EBDM Capacity	0.25	2.12	0.15	1.29 (0.92, 1.81)	−0.59	7.26	0.007	0.55 (0.36, 0.85)
Resource Availability	0.32	2.68	0.10	1.38 (0.94, 2.20)	−0.71	8.51	0.004	0.49 (0.30, 0.79)
Evaluation Capacity	0.27	2.98	0.08	1.31 (0.96, 1.79)	−0.67	12.06	0.005	0.51 (0.35, 0.75)
Climate Cultivation	0.36	2.74	0.10	1.44 (0.94, 2.21)	−0.96	12.10	0.005	0.39 (0.23, 0.66)
Partnerships that Support EBDM	0.15	0.57	0.45	1.16 (0.79, 1.69)	−0.48	4.37	0.04	0.62 (0.39, 0.97)
EBDM support overall (sum of 6)	−0.07 (0.50)	0.02	0.89	1.06 (0.99, 1.14)	−0.15 (0.05)	10.93	< 0.001	0.86 (0.79, 0.94)

^a^A separate model was conducted for each mis-implementation type (dependent variable) and each EBDM factor (independent variable of interest)

^b^ENDING models were adjusted for: Jurisdiction population size, state, having a local board of health, having a graduate degree in any field, having a nursing background, and years worked in public health

^c^CONTINUING models were adjusted for jurisdiction population size, state, having a graduate degree in public health, and age group

### Reasons for mis-implementation

**Fig. 1:**
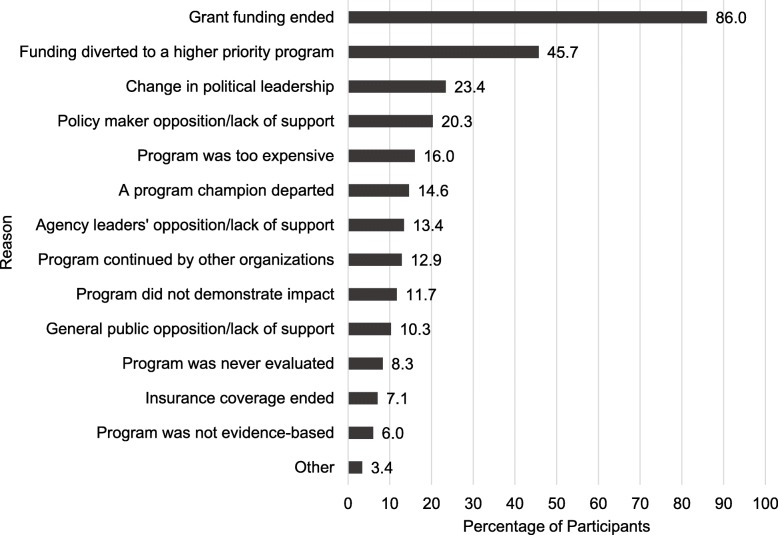
Reasons for ENDING programs that should have continued in local health departments, *n* = 350. Legend: Total percent does not equal 100% as participants could select more than one option

Figure 1 shows percentages of participants that selected pre-listed potential reasons for inappropriate termination. The most commonly chosen reasons were the ending of funding, either that grant funding ended (86.0%), or that funding was diverted to a higher priority program (45.7%). Fewer than 25% of participants selected each of the remaining pre-listed reasons. Twelve participants (3.4%) reported “other” reasons, which included lack of staff (*n* = 4), other funding issues (*n* = 4), low program participation (*n* = 2), and single responses of other reasons.

**Fig. 2:**
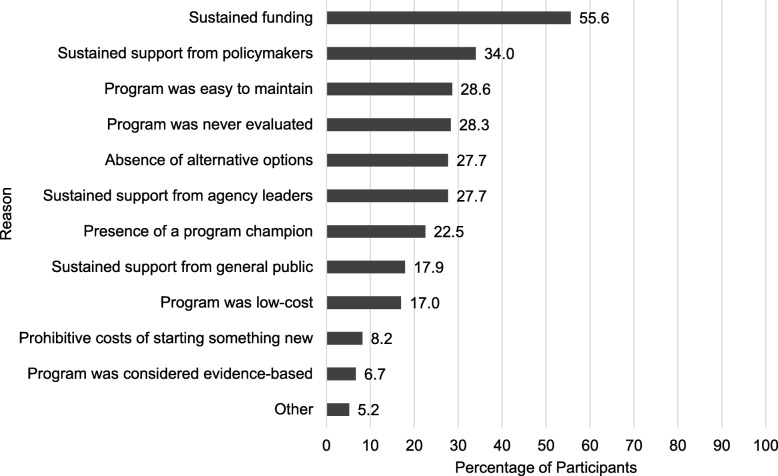
Reasons for CONTINUING programs that should have ended in local health departments, *n* = 329. Legend: Total percent does not equal 100% as participants could select more than one option

As shown in Fig. 2, the most frequently selected reasons chosen for inappropriate continuation were sustained funding (55.6%) and sustained support from policymakers (34.0%). Sustained support from agency leaders (27.7%), ease of maintaining the program (28.6%), lack of program evaluation (28.3%), and absence of alternative program options (27.7%) were also cited by more than 25%. The 17 (5.2%) “other” responses included resistance to change (*n* = 8); and continuation was “required”, “mandated”, “requested”, or “supported by others” (*n* = 6). Resistance to change included “it’s what we’ve always done”, “inertia”, “tradition”, “staff ingrained”, “fear of change”, and “resistance to stopping”. Reasons did not vary by reported frequency of inappropriate continuation (data not shown).

## Discussion

Our study shines light on two different phenomena in mis-implementation of public health programs among local public health practice: inappropriate termination and inappropriate continuation. For programs ending when they should have continued, most reported funding ending as the main reason, while support for EBDM within the LHD was not a factor in frequency. For programs continuing when they should have ended, the most common reason was that funding was sustained. Furthermore, in this instance there appeared to be an association between a lack of organizational support for EBDM and inappropriate continuation.

Reported frequency of mis-implementation in this study was lower than that found in the study team’s earlier pilot work [2], but still concerning. In pilot data from 2013 to 2014 with identical item wording, 42.0% of LHD directors and program managers reported programs often or always ended that should have continued and 29.4% reported programs often or always continued that should have ended [2], compared to 30.0 and 16.4% respectively in the present study. Sampling methods differed, so findings may not be fully comparable. Nonetheless, the lower reported frequencies in the present study may reflect funders’ increased requirements since the previous study for LHDs to demonstrate use of EBIs in chronic disease prevention. Given current national emphasis on EBIs, there may also have been reluctance to report inappropriate continuation (social desirability bias).

It is encouraging that higher perceived organizational supports for EBDM were associated with lower inappropriate continuation of programs, but it is puzzling that several organizational support factors trended toward positive non-significant associations with inappropriate continuation. We can only surmise that managers in LHDs with higher evaluation capacity may be more aware of inappropriate termination. As shown in Fig. 1, only 8% reported lack of evaluation, and only 12% reported lack of program impact, as top reasons for inappropriate termination.

Organizational supports were insufficient in this study to ensure program sustainment, while other studies found multiple internal and external factors affected sustainment. Reviews found organizational climate, leadership support, staff commitment and skills, adequate staffing and low staff turnover, organizational resources, and partnerships affect EBI sustainability [18, 45, 46]. The review by Hodge et al. (2016) found engagement with community leaders and individual implementers key to community-based program sustainment in low resource settings [18]. Engagement involves building relationships with community policy makers and implementers [18, 46]. An important aspect is making decisions together to ensure programs are aligned with community context, cultures, and priorities [18, 46]. Collaborative partnerships across organizations and coalitions are also key to program sustainment [18, 45, 46]. High functioning organizational partnerships that leverage capacity of each collaborating organization are more likely to be able to sustain programs [18, 45, 46]. Policy and legislation are associated with sustainment of programs in community and clinical and social service settings [45]. Engaging community leaders and other policy makers throughout programmatic decision-making can increase likelihood of program sustainment [18].

Qualitative studies emphasize the importance of leadership support, planning, partnerships, and communication in capacity to sustain public health EBIs [26, 47]. Reassignment of staff, changes in staff workloads, and changes in leadership led to discontinuation of an evidence-based trauma intervention in a large urban school district [48]. Lack of organizational policy to support staff time to attend training led to partial implementation of after school physical activity efforts [49]. But in the present study, ending of funding was by far the most commonly reported reason for inappropriate termination, as found in a recent review [50], and organizational supports were not protective. This reflects lack of ongoing funding sources for EBIs, and points out the need for strong community and policy maker engagement, inter-organizational partnerships, and alternate and diversified funding sources. There is a need for better communication with policy makers and other program decision makers on the importance of and evidence for population-based chronic disease prevention. Communicating evidence to policy makers remains one of the top skill gaps among health department staff [51].

Public health systems are working to scale up EBIs [22, 45, 49], but little is known about strategies to address inappropriate continuation of ineffective approaches. Here public health can learn from medical studies, even though organizational structures and funding sources differ. Healthcare systems are acknowledging that ending low value care is difficult, requires different processes than implementation, and best strategies are not yet known [9]. Supportive organizational climates and training are two organizational supports that may help. A recent review by Colla and colleagues found healthcare systems that provided clinician education and feedback decreased use of low value procedures [7], but other authors viewed provider education and feedback as insufficient [9, 10, 52]. For example, clinician awareness of guidelines to avoid use of low-value tumor markers did not lead to ending such use except in healthcare systems with cultures that emphasized collective decision making in line with guidelines [52].

The present study has several limitations. The cross-sectional survey limits temporal interpretations of the findings. We do not know how public health practitioners perceive programs as something that should continue or end. However, these questions were asked after detailed definition of EBDM and sections on EBIs and supports for EBDM. Participants may have different interpretations of the terms “evidence-based” and “effectiveness.” We did not define the term “warranted” in the questions “In your opinion, how often do programs end that should have continued (i.e., end without being warranted)”; and “In your opinion, how often do programs continue that should have ended (i.e., continue without being warranted). So it is unknown whether participants interpreted “warranted” as evidence-based, which was the context of the survey, or were mentally including other factors such as champion or policy maker preferences or lack of partner support. We also did not specify a time frame for perceived frequency of inappropriate termination or continuation. There was not space in the survey to ask participants to define what they meant by inappropriate program termination or continuation, which would have furthered understanding and interpretation of survey responses. There could be social desirability bias to under-report continuation of programs that should end, given national emphasis on EBIs for chronic disease prevention. Still, the present study provides a glimpse into mis-implementation in local public health. A future study will address many of these limitations [3].

In addition to gaining a deeper understanding of organizational and external influences on mis-implementation, future solutions need to be developed for how best to fund public health practice so that effective programs can be sustained. With high staff turnover and a significant portion of the public health workforce retiring [16, 17], succession planning and ongoing EBDM capacity building efforts are needed.

## Conclusions

While improvements have occurred since early pilot data were collected in 2013 [2], the results of this study show that both inappropriate termination and continuation of programs continue, mainly due to funding-related issues. Loss of funding was the main reason reported for inappropriate termination, with organizational supports not protective. Policy maker engagement, strong organizational partnerships, and creative mechanisms of support are needed to avoid inappropriate termination. This study shows organizational supports for EBDM may help LHDs avoid inappropriate continuation, but may be secondary to funding considerations. Public health agency leaders can cultivate organizational climates in which EBDM is valued and supported; ensure staff are trained in skills needed for EBDM, including program implementation and evaluation; provide access to evidence; and stimulate strong partner networks. Further understanding of the local and national influences on mis-implementation among LHDs can help identify financial and other supports so resources can be directed towards programs with the greatest promise of improving health and well-being.

## Supplementary information

**Additional file 1.**

## Data Availability

Materials related to this study are available on the Prevention Research Center in St. Louis at Washington University in St. Louis website https://prcstl.wustl.edu/. The datasets analyzed during the current study are available from the corresponding author on reasonable request.

## Abbreviations

CDC: Centers for Disease Control and Prevention
D.A.R.E.: Drug Abuse Resistance Education program
EBDM: Evidence-based decision making
HIV: Human immunodeficiency virus
ICC: Intra-cluster correlation
LHD: Local health department
NACCHO: National Association of County and City Health Officials
