# Supplementary material for: Perspectives on program mis-implementation among U.S. local public health departments
Source: BMC Health Serv Res. 2020 Mar 30;20:258. doi: 10.1186/s12913-020-05141-5 (PMC7106610; doi:10.1186/s12913-020-05141-5)
Supplement: Supplementary file 1 — Additional file 1. [file 12913_2020_5141_MOESM1_ESM.pdf]

Adoption and Implementation of evidence to Mobilize Local Health (AIM-Local Health)
Dissemination Survey for Lead Chronic Disease Practitioners at LHDs of varying size across U.S.

Domains	# of Items	Item sources
Background	9	[1]
Interventions to address chronic diseases	8	[2-4]
Views on evidence-based interventions	4	[1,5]
Health equity	2	New
Evidence-based decision making (EBDM) definitions and supports	2	[5,6]
Importance and Availability of EBDM	20	[7,8]
EBDM Dissemination stages	22	[9]
Administrative evidence-based practices (org culture and climate-1, relationships and partnerships-4)	5	[10,11]
Ending programs/mis-implementation	4	[12]
Work style (Grit short scale)	8	[13] New in the public health context
Academic health department partnerships	2	New (from Paul Erwin – current project studying characteristics AHD partnerships from PH schools' or programs' perspective)
**Total**	**86**	

Purpose: Ross Brownson and colleagues at the Prevention Research Center at Washington University in St. Louis are working on a new project funded by the National Institutes of Health. We are exploring strategies to support the use of evidence-based diabetes and chronic disease prevention and control among local health departments. We are asking public health practitioners to help us by completing this survey. We will use the findings to plan ways to support use of public health knowledge in diabetes and chronic disease control.

This survey will take approximately 20 minutes of your time. Your responses are anonymous and participation is voluntary. Survey responses are not linked to anyone's name or email address. Only summary findings will be reported that do not identify people or organizations.

To thank you for your time, we are offering everyone who completes the survey an optional $20 Amazon gift card. The gift card is not required to participate in the survey. At the end of the survey, you will be redirected to an optional separate webpage where you can choose to accept the gift card.

I understand my participation in this survey is voluntary, and my answers are anonymous.
- ❍ Yes, I would like to take this survey
- ❍ No, I prefer not to take this survey [If selected, skip to the end of the survey.]

Adoption and Implementation of evidence to Mobilize Local Health (AIM-Local Health)
Dissemination Survey for Lead Chronic Disease Practitioners at LHDs of varying size across U.S.

SECTION A: BACKGROUND
Please answer the following questions about your background.

1. Which of the following best describes your position?

    a. Top executive, health director, health officer, commissioner, or equivalent in the "office of the director"
    b. Administrator, deputy or assistant director
    c. Manager of a division or program
    d. Program coordinator
    e. Technical expert position (evaluator, epidemiologist, health educator)
    f. Other (please specify)

2. How long have you been in your current position?
    Years

3. Over your whole career how long have you been involved in public health?
    Years

4. Which degree/credentials do you hold (check all that apply)?
    Associate degree
    BS/BA
    CHES
    Certified in Public Health
    LPN
    RN
    BSN
    ARNP (Advanced Registered Nurse Practitioner)
    RD
    MS or MSc
    MPH or MSPH
    MSW
    MPA
    MA
    Other Masters Degree
    MD or DO
    PhD, DrPH, or ScD in a public health field
    PhD or ScD in another field
    EdD
    RS (Registered Sanitarian)
    Other (please specify below) ____________________

5. Are you…?
    Male
    Female
    Other
    Prefer not to answer

6. What is your race? (Check all that apply)
   - White
   - Black/African-American
   - American Indian, Alaskan Native
   - Asian
   - Native Hawaiian or Other Pacific Islander
   - Hispanic or Latino
   - Other
   - Prefer not to answer

7. What is your age?
   - 20-29 years
   - 30-39 years
   - 40-49 years
   - 50-59 years
   - 60 years or older

8. Is your health department accredited or preparing to apply for accreditation through the Public Health Accreditation Board (PHAB)?
   - We are currently accredited
   - Yes, and we have recently applied but are not yet accredited
   - Yes, but we have not yet applied
   - No
   - Unsure

9. Please indicate if you work in the below program areas by answering Yes or No.

a. Diabetes	Yes	No
b. Obesity	Yes	No
c. Physical Activity	Yes	No
d. Nutrition	Yes	No
e. Tobacco	Yes	No

[Program Qualtrics to select topics or items based on the decision logic below. This way no one will be asked more than 4 items.]
If participant answers YES to only one program area, then Qualtrics to select the respective program area interventions.
If participant answers NO to all program areas, then Qualtrics to randomly select one program area's interventions of the 5 program areas.
If Diabetes is selected with other program areas, the Qualtrics to select diabetes interventions.
If Diabetes is not selected but multiple program areas are selected, then Qualtrics randomly selects one of the selected program areas' interventions.

SECTION B: INTERVENTIONS TO ADDRESS CHRONIC DISEASES

You will read about various programs, policies and services intended to prevent or address chronic diseases. For each item, please indicate whether or not your agency has directly delivered each intervention, AND if your agency collaborated with community organizations to support the delivery of each intervention by responding "yes, no, or don't know." No single agency is expected to have implemented all the following interventions.

Hover your mouse over blue text for definition.

10. In the past year, has your agency* directly delivered, and has your agency collaborated with organizations to support delivery of the following ***diabetes*** interventions?
[*Rollover definition – **agency:** the district, county or city local public health agency in which you are employed.]

	**Has your agency* directly delivered/provided?**	**Has your agency* collaborated with* organization(s) to support delivery?** [rollover definition: served as a community/clinical referral source, or a convener that facilitates the program and referral systems]
a. Diabetes prevention program/DPP - diet and physical activity promotion programs with people at increased risk for type 2 diabetes.	Yes No Don't know	Yes No Don't know
b. Community health workers* to deliver diet and physical activity promotion, and weight management to groups or individuals with increased risk for type 2 diabetes. [*Rollover definition: frontline, trained staff who are from or have a close understanding of the community served to bridge healthcare]	Yes No Don't know	Yes No Don't know
c. Diabetes self-management education with persons with diabetes delivered in community gathering places.	Yes No Don't know	Yes No Don't know
d. Diabetes management: identifying patients with diabetes and determining effective treatment.	Yes No Don't know	Yes No Don't know

11. In the past year, has your agency* directly delivered, and has your agency collaborated with organizations to support delivery of the following ***obesity*** interventions?

	**Has your agency* directly delivered/provided?**	**Has your agency* collaborated with* organization(s) to support delivery?**
a. Worksite programs, policies or environmental changes to support nutrition/healthy food and physical activity.	Yes No Don't know	Yes No Don't know
b. Behavioral interventions to reduce screen time among children OR reduce screen time plus increase physical activity/healthy	Yes No Don't know	Yes No Don't know

eating.		
c. Multicomponent interventions with coaching that uses technology to communicate with individuals or groups to help them lose or maintain weight (e.g., pedometers with computer interaction, social media).	Yes No Don't know	Yes No Don't know
d. Obesity screening and management: screening adults and referring patients with a body mass index (BMI) of 30 kg/m2 or higher to behavioral interventions	Yes No Don't know	Yes No Don't know

12. In the past year, has your agency* directly delivered, and has your agency collaborated with organizations to support delivery of the following ***physical activity*** interventions?

	**Has your agency* directly delivered/provided?**	**Has your agency* collaborated with* organization(s) to support delivery?**
a. Programs that set up social support for physical activity (e.g. walking groups, buddy/accountability systems)	Yes No Don't know	Yes No Don't know
b. Programs, policies, or environmental changes that make streets safer for pedestrians and cyclists (e.g. street lighting, crosswalks, bike lanes, Complete Streets, Safe routes to Schools)	Yes No Don't know	Yes No Don't know
c. Programs or policies that create or improve access to places for physical activity (e.g., walking trails, making exercise facilities more accessible, joint use agreements between schools and communities).	Yes No Don't know	Yes No Don't know
d. Programs or policies that increase physical activity in schools (e.g., time in school PE classes, training for PE teachers, equipment and materials, physical activity breaks).	Yes No Don't know	Yes No Don't know

13. In the past year, has your agency* directly delivered, and has your agency collaborated with organizations to support delivery of the following ***nutrition*** interventions?

	**Has your agency* directly delivered/provided?**	**Has your agency* collaborated with* organization(s) to support delivery?**
a. Policies or environmental changes to improve access to healthy foods in worksites, schools, or other local facilities (e.g., changing cafeteria options, vending machine content, healthy meeting meals)	Yes No Don't know	Yes No Don't know
b. Policies or changes that improve healthier food choices through nutrition assistance programs (e.g., WIC, SNAP, Senior Nutrition Programs).	Yes No Don't know	Yes No Don't know
c. School gardens that allow students to garden during school or non-school hours with school staff guidance.	Yes No Don't know	Yes No Don't know
d. Policies, environmental changes or programs promoting breastfeeding initiation, exclusive breastfeeding, and duration of breastfeeding.	Yes No Don't know	Yes No Don't know

14. In the past year, has your agency* directly delivered, and has your agency collaborated with organizations to support delivery of the following ***tobacco control*** interventions?

	**Has your agency* directly delivered/provided?**	**Has your agency* collaborated with* organization(s) to support delivery?**
a. Reminders for clinic healthcare providers to discuss tobacco/nicotine cessation with clients (e.g. chart stickers, medical record check lists)	Yes No Don't know	Yes No Don't know
b. Mass health communication with cessation messages AND quitline number.	Yes No Don't know	Yes No Don't know
c. Public education about clean indoor air policies or the expansion of these policies	Yes No Don't know	Yes No Don't know
d. Public education about the effects of tobacco unit price on preventing and reducing tobacco consumption	Yes No Don't know	Yes No Don't know

SECTION C: YOUR VIEWS ON EVIDENCE-BASED INTERVENTIONS

This section asks about your views on **evidence-based interventions,** which are programs, policies, and services with evidence (based on published research) of improving health, such as those covered in the previous section. This refers to evidence-based strategies, like behavioral changes, changes to the physical environment, policy approaches, or clinical services that are specific and structured. Please indicate the extent to which you agree or disagree with the following statements.

15. Evidence-based interventions* are appropriate and relevant to the specific populations my agency serves. [*Definition rollover: Policies, programs, and practices that are based on the best available public health science, knowledge, and data; refers to behavioral strategies, changes to the physical environment, policy approaches, or clinical services that specific and structured]

Strongly Disagree 1 2 3 4 5 6 7 Strongly Agree

16. I have the skills I need to adapt evidence-based interventions* from one setting or population to another. [*Definition rollover – same as above]

Strongly Disagree 1 2 3 4 5 6 7 Strongly Agree

17. Program staff in my work group/division* (rollover definition) is aware of published evidence reviews of interventions (e.g., The Community Guide, Cochrane Reviews).

Strongly Disagree 1 2 3 4 5 6 7 Strongly Agree

18. Program staff in my work group/division is aware of toolkits for planning and evaluation.

Strongly Disagree 1 2 3 4 5 6 7 Strongly Agree

19. Do staff in your work group/division implement interventions to achieve health equity*?
[*Definition rollover - **health equity**: Ensuring all people have the opportunity to reach their highest level of health.]

Yes [If "Yes," go to Q20]
No [Skip to Q21]
Not Sure [Skip to Q21]

20. [If "Yes" to Q19] If possible, please share an example – an intervention your work group/division has implemented in the past 12 months to achieve health equity. [NOT REQUIRED]
Text response

SECTION D: EVIDENCE-BASED DECISION MAKING DEFINITIONS AND SUPPORTS

Now we are shifting away from specific interventions to a broader framework on use of an ***evidence-based process*** to plan and carry out public health programs, policies and practices in chronic disease control. Throughout the remainder of the survey, *such a process is referred to as **evidence-based decision-making*** and involves:

- Making decisions based on the best available scientific and/or rigorous program evaluation evidence;
- Applying program planning and quality improvement frameworks;
- Engaging the community in assessment and decision making;
- Adapting and implementing evidence-based interventions for specific populations or settings; and
- Conducting sound evaluation – the process of determining the relevance, effectiveness, and impact of activities according to their goals.

21. Within your agency, which of the following would most encourage you to utilize evidence-based decision-making* in your work? Using the list below, please rank the top three, where 1 is the most important. [Program Qualtrics for drag and drop]
[*Prioritizing issues and implementing interventions based on sound science combined with community engagement, and evaluation.]

Leaders in my agency placing a high priority on evidence-based decision-making
Direct supervisors placing a high priority on evidence-based decision-making
Positive feedback or encouragement to use evidence-based decision-making
Easy access to data resources for evidence-based decision-making
Support for travel to regional or national trainings or meetings *to learn about* evidence-based decision-making
An employee performance evaluation that supports the use of evidence-based decision-making
Professional recognition for use of evidence-based decision-making
Making evidence-based decision-making part of job descriptions in my agency
Support for travel to regional or national trainings or meetings *to present on* evidence-based decision-making
Other: please specify

22. Which of the following would be most useful to you in building skills for evidence-based decision-making* in your work? From the items below, please rank the top three, where 1 is the most useful.

General evidence-based decision-making training workshops
Evidence-based decision-making training for specific program areas
Access to experts by phone or email for questions about evidence-based decision-making
Help with evidence-based decision-making processes (e.g. community assessment, evaluation)
Evidence-based decision-making webinars
Self-paced evidence-based decision-making internet programs or trainings
Interactive evidence-based decision-making web-based meetings

Learning to write summaries of research evidence (issue briefs)
Learning to write policy briefs
Network of health departments and agencies that meets online
Network of health departments and agencies that meets in person
Other: please specify

SECTION E: IMPORTANCE AND AVAILABILITY OF EVIDENCE-BASED DECISION MAKING

Now, we would appreciate your help rating the importance and availability of each skill in the statements below. First, read the statements (skills in evidence-based decision-making) below; then, use the first scale to rate the importance of each of the skills to you. Next, use the second scale to rate how available each skill is to you when you need it (either in your own skill set or among others in your agency).

23. Community assessment: Understand how to describe the health issue according to the needs and assets of the population/community of interest.

Unimportant	0	1	2	3	4	5	6	7	8	9	10	Very Important
Not Available	0	1	2	3	4	5	6	7	8	9	10	Very Available

24. Quantifying the issue: Understand the uses of descriptive epidemiology (i.e., compiling and analyzing data by person, place, and time) in quantifying a public health issue.

Unimportant	0	1	2	3	4	5	6	7	8	9	10	Very Important
Not Available	0	1	2	3	4	5	6	7	8	9	10	Very Available

25. Prioritization: Understand how to prioritize program and policy options.

Unimportant	0	1	2	3	4	5	6	7	8	9	10	Very Important
Not Available	0	1	2	3	4	5	6	7	8	9	10	Very Available

26. Action planning: Understand the importance of developing an action plan for how to achieve goals and objectives.

Unimportant	0	1	2	3	4	5	6	7	8	9	10	Very Important
Not Available	0	1	2	3	4	5	6	7	8	9	10	Very Available

27. Adapting interventions: Understand how to modify programs and policies for different communities and settings.

Unimportant	0	1	2	3	4	5	6	7	8	9	10	Very Important
Not Available	0	1	2	3	4	5	6	7	8	9	10	Very Available

28. Evaluation designs: Understand the different designs that are useful in program or policy evaluation (i.e., quasi-experimental designs).

Unimportant	0	1	2	3	4	5	6	7	8	9	10	Very Important
Not Available	0	1	2	3	4	5	6	7	8	9	10	Very Available

29. Quantitative evaluation: Understand the uses of quantitative evaluation approaches (e.g., surveillance and/or surveys).

Unimportant	0	1	2	3	4	5	6	7	8	9	10	Very Important
Not Available	0	1	2	3	4	5	6	7	8	9	10	Very Available

30. Qualitative evaluation: Understand the value of qualitative evaluation approaches (e.g., focus groups, key informant interviews) including the steps involved in conducting qualitative evaluations.

Unimportant	0	1	2	3	4	5	6	7	8	9	10	Very Important
Not Available	0	1	2	3	4	5	6	7	8	9	10	Very Available

31. Economic evaluation: Understand how to use economic data (e.g., cost-effectiveness) in the decision making process.

Unimportant	0	1	2	3	4	5	6	7	8	9	10	Very Important
Not Available	0	1	2	3	4	5	6	7	8	9	10	Very Available

32. Communicating evidence to decision-makers: Understand the importance of effectively communicating with decision-makers about public health issues (e.g., elected officials, superintendents, business leaders, or other key community partners).

Unimportant	0	1	2	3	4	5	6	7	8	9	10	Very Important
Not Available	0	1	2	3	4	5	6	7	8	9	10	Very Available

SECTION F: SPREADING EVIDENCE-BASED DECISION MAKING

The next sections of the survey will help us understand readiness to support use of evidence-based decision-making*. (*EBDM rollover) For the following statements, please indicate the extent to which you agree or disagree. Please remember that your responses are anonymous and only summary findings that do not identify people or organizations will be reported.

*[Definition rollover – evidence-based decision-making: Prioritizing issues and implementing interventions based on sound science combined with community engagement, sound management, and evaluation.]

Hover your mouse over blue text for definition.

Awareness

33. I am provided the time to identify evidence-based programs and practices.

Strongly Disagree	1	2	3	4	5	6	7	Strongly Agree

34. My direct supervisor recognizes the value of management practices* (provide rollover) that facilitate evidence-based decision-making.
    [*For example, asking for employee input, hiring well-trained staff, supporting learning, building partnerships, and using effective budget practices.]

Strongly Disagree	1	2	3	4	5	6	7	Strongly Agree

35. My work group/division* offers employees opportunities to attend evidence-based-decision making trainings. [rollover work group/division definition]

Strongly Disagree 1 2 3 4 5 6 7 Strongly Agree

36. Top leadership in my agency (e.g., director, assistant directors) recognizes the value of evidence-based decision-making.

Strongly Disagree 1 2 3 4 5 6 7 Strongly Agree

Use of EBDM

37. I use evidence-based decision making in my work.

Strongly Disagree 1 2 3 4 5 6 7 Strongly Agree

38. My direct supervisor expects me to use evidence-based decision making.

Strongly Disagree 1 2 3 4 5 6 7 Strongly Agree

39. My performance is partially evaluated on how well I use evidence-based decision making in my work.

Strongly Disagree 1 2 3 4 5 6 7 Strongly Agree

40. My work group/division* currently has the resources (e.g. staff, facilities, partners) to support application of evidence-based decision making.

Strongly Disagree 1 2 3 4 5 6 7 Strongly Agree

41. The staff in my work group/division* has the necessary skills to carry out evidence-based decision making.

Strongly Disagree 1 2 3 4 5 6 7 Strongly Agree

42. The majority of my work group/division's* external partners support use of evidence-based decision making.

Strongly Disagree 1 2 3 4 5 6 7 Strongly Agree

43. Top leadership in my agency encourages use of evidence-based decision making.

Strongly Disagree 1 2 3 4 5 6 7 Strongly Agree

Resource Maintenance

44. Informational resources (e.g. academic journals, guidelines, and toolkits) are available to my work group/division* to promote the use of evidence-based decision making.

Strongly Disagree 1 2 3 4 5 6 7 Strongly Agree

45. My work group/division* engages a diverse external network of partners that share resources* to facilitate evidence-based decision making. [rollover resources for EBDM examples: For example, staff, facilities, data, toolkits, journal articles, access to training]

Strongly Disagree 1 2 3 4 5 6 7 Strongly Agree

46. Stable funding is available for evidence-based decision making.

Strongly Disagree 1 2 3 4 5 6 7 Strongly Agree

Evaluation Maintenance

47. My work group/division* supports community needs assessments to ensure that evidence-based decision making approaches continue to meet community needs.

Strongly Disagree 1 2 3 4 5 6 7 Strongly Agree

48. My work group/division plans for evaluation of interventions prior to implementation.

Strongly Disagree 1 2 3 4 5 6 7 Strongly Agree

49. My work group/division uses evaluation data to monitor and improve interventions.

Strongly Disagree 1 2 3 4 5 6 7 Strongly Agree

50. My work group/division distributes intervention evaluation findings to other organizations that can use our findings.

Strongly Disagree 1 2 3 4 5 6 7 Strongly Agree

Organizational Climate

51. My work group/division* has access to evidence-based decision making information that is relevant to community needs.

Strongly Disagree 1 2 3 4 5 6 7 Strongly Agree

52. When decisions are made within my work group/division*, program staff members are asked for input.

Strongly Disagree 1 2 3 4 5 6 7 Strongly Agree

53. Information is widely shared in my work group/division* so that everyone who makes decisions has access to all available knowledge.

Strongly Disagree 1 2 3 4 5 6 7 Strongly Agree

54. My agency is committed to hiring people with relevant training or experience in public health core disciplines (e.g. epidemiology, health education, environmental health).

Strongly Disagree 1 2 3 4 5 6 7 Strongly Agree

55. My agency has a culture that supports the processes necessary for evidence-based decision making.

Strongly Disagree 1 2 3 4 5 6 7 Strongly Agree

Relationships and Partnerships

56. Our collaborative partnerships have missions that align with my agency.

Strongly Disagree 1 2 3 4 5 6 7 Strongly Agree

57. It is important to my agency to have partners who share resources (money, staff time, space, materials).

Strongly Disagree 1 2 3 4 5 6 7 Strongly Agree

58. It is important to my agency to have partners in healthcare to address population health issues.

Strongly Disagree 1 2 3 4 5 6 7 Strongly Agree

59. It is important to my agency to have partners in other sectors (outside of health) to address population health issues.

Strongly Disagree 1 2 3 4 5 6 7 Strongly Agree

SECTION F: ENDING PROGRAMS

60. When you think about public health programs that have ended when they should have continued, what are the most common reasons for programs ending? (*Select the top three*)

Program was never evaluated
Program was evaluated but did not demonstrate impact
Opposition/lack of support from leaders in my agency
Opposition/lack of support from the general public
Opposition/lack of support from policy makers
Funding diverted to a higher priority program
Grant funding ended
Change in political leadership
Insurance funding/coverage ended
Program was adopted or continued by other organizations
A program champion departed
Program was not evidence-based
Program was too expensive
Other, please specify ______
I do not know
Not applicable

61. In your opinion, how often do programs end that should have continued? (i.e., end without being warranted) *(select one)*

    Never
    Rarely
    Sometimes
    Often
    Always
    I do not know
    Not applicable

62. When you think about public health programs that continued when they should have ended, what are the most common reasons for their continuation? (i.e., continue without being warranted) (*Select the top three*)

    Program was never evaluated
    Sustained support from leaders in your agency
    Sustained support from the general public
    Sustained support from policymakers
    Prohibitive costs of starting something new
    Absence of alternative options
    Sustained funding
    Presence of a program champion
    Program was considered evidence-based
    Program was low-cost
    Program was easy to maintain
    Other, please specify ______
    I do not know
    Not applicable

63. In your opinion, how often do programs continue that should have ended? (i.e., continue without being warranted) *(select one)*

    Never
    Rarely
    Sometimes
    Often
    Always
    I do not know
    Not applicable

SECTION H: YOUR WORK STYLE

64. This next section asks about your work style and how passionate and persevering you see yourself to be. Below are a number of statements that may or may not apply to you. There are no right or wrong answers, so just answer honestly, considering how you compare to most people. Please remember your responses are anonymous and only summary findings that do not identify people or organizations will be reported.

	Not Like Me At All (1)	Not Much Like Me (2)	Somewhat Like Me (3)	Mostly Like Me (4)	Very Much Like Me (5)
New ideas and projects sometimes distract me from previous ones. (1)	❍	❍	❍	❍	❍
Setbacks don't discourage me. I don't give up easily. (2)	❍	❍	❍	❍	❍
I have been strongly focused on a certain idea or project for a short time but later lost interest. (3)	❍	❍	❍	❍	❍
I am a hard worker. (4)	❍	❍	❍	❍	❍
I often set a goal but later choose to pursue a different one. (5)	❍	❍	❍	❍	❍
I have difficulty maintaining my focus on projects that take more than a few months to complete. (6)	❍	❍	❍	❍	❍
I finish whatever I begin. (7)	❍	❍	❍	❍	❍
I am diligent. (8)	❍	❍	❍	❍	❍

SECTION I: ACADEMIC PARTNERSHIPS

The questions in this section inquire about academic partnerships. These are partnerships between a university, college, or other academic institution and a governmental public health agency that provides mutual benefits in teaching, research, and service. It may be a formal agreement, i.e., a signed memorandum of understanding, a letter, or agreement that outlines and directs the activities of the academic institution's faculty, staff, and students and the governmental public health agency's staff.

65. Does your agency currently participate in any academic partnerships*?
    [*Definition rollover: Arrangement between an academic institution and a governmental public health agency that provides mutual benefits in teaching, research, and service.]

    Yes [If "YES", skip to Q67]
    No [If "No" or "Unsure", skip to Q66]
    Unsure

66. [If "No" or "Unsure" to Q65] Why does your agency not participate in an academic partnership?

    I am not familiar or never heard of such a partnership
    Time constraints
    Resource constraints
    Difficulty establishing a connection with an academic department/school
    Location/distance of university from our agency
    Difficulty obtaining approval from agency leadership
    Other (Please specify)

67. Which of the following characteristics describe your agency's relationships with academic institutions (check all that apply)?

Formal written partnership agreement(s)
Shared staff
Shared financial resources
Compensation for services provided
Public health training and educational opportunities
Hosting student interns
Joint research projects
Shared provision of public health services
Shared facilities
University library access for health department staff
Unsure
Other (Please specify)

THANK YOU!

Thank you for completing this survey! Your time, efforts, and responses are greatly appreciated by all of us at the Prevention Research Center in St. Louis. Your efforts today will help us plan how best to support local health agencies as we move forward.

If you have questions, please call Renee Parks at 314-935-0148 or email: renee.parks@wustl.edu

To request a summary of project findings, or to accept your optional $20 Amazon gift card, please go to the following link:

It is a separate website where you will be asked to give your e-mail address. If you choose to accept the gift card you will also be asked personal information so that Washington University in St. Louis can process and send your gift card to you. The information cannot be traced to your survey responses and will be kept confidential.

SURVEY REFERENCES

1. Jacobs JA, Dodson EA, Baker EA, Deshpande AD, Brownson RC. Barriers to Evidence-Based Decision Making in Public Health: A National Survey of Chronic Disease Practitioners. *Public Health Reports.* Sep-Oct 2010;125(5):736-742.
2. Community Preventive Services Task Force. The Guide to Community Preventive Services. www.thecommunityguide.org. Accessed October 19, 2016.
3. University of Wisconsin Population Health Institute. What Works for Health Policies and Programs to Improve Wisconsin's Health. http://whatworksforhealth.wisc.edu/. Accessed October 19, 2016.
4. US Preventive Services Task Force. Guide to Clinical Preventive Services. 2014; 4th:https://www.uspreventiveservicestaskforce.org/. Accessed February 20, 2017 2017.
5. Reis RS, Duggan K, Allen P, Stamatakis KA, Erwin PC, Brownson RC. Developing a Tool to Assess Administrative Evidence-Based Practices in Local Health Departments. *American journal of public health.* 12/ 2014;104(12):e43-e43.
6. Jacobs JA, Clayton PF, Dove C, et al. A survey tool for measuring evidence-based decision making capacity in public health agencies. *BMC Health Services Research.* 2012;12:57-57.

7. Brownson RC, Ballew P, Kittur ND, et al. Developing competencies for training practitioners in evidence-based cancer control. *J Cancer Educ.* 2009;24(3):186-193.
8. Jacob RR, Baker EA, Allen P, et al. Training needs and supports for evidence-based decision making among the public health workforce in the United States. *BMC Health Services Research.* 2014;14(1):564.
9. Stamatakis KA, Ferreira Hino AA, Allen P, et al. Results from a psychometric assessment of a new tool for measuring evidence-based decision making in public health organizations. *Eval Program Plann.* Feb 2017;60:17-23.
10. Brownson RC, Allen P, Duggan K, Stamatakis KA, Erwin PC. Fostering more-effective public health by identifying administrative evidence-based practices: a review of the literature. *Am J Prev Med.* Sep 2012;43(3):309-319.
11. Brownson RC, Reis RS, Allen P, et al. Understanding administrative evidence-based practices: findings from a survey of local health department leaders. *Am J Prev Med.* Jan 2013;46(1):49-57.
12. Brownson RC, Allen P, Jacob RR, et al. Understanding mis-implementation in public health practice. *Am J Prev Med.* May 2015;48(5):543-551.
13. Duckworth AL, Quinn PD. Development and validation of the short grit scale (grit-s). *Journal of personality assessment.* Mar 2009;91(2):166-174.
